# The prevalence and distribution of the amyloidogenic transthyretin (*TTR*) V122I allele in Africa

**DOI:** 10.1002/mgg3.231

**Published:** 2016-07-14

**Authors:** Daniel R. Jacobson, Alice A. Alexander, Clement Tagoe, W. T. Garvey, Scott M. Williams, Sara Tishkoff, David Modiano, Sodiomon B. Sirima, Issa Kalidi, Amadou Toure, Joel N. Buxbaum

**Affiliations:** ^1^ Veterans Administration Boston Healthcare System and Department of MedicineBoston University School of MedicineBostonMassachusetts; ^2^ Research ServiceVeterans Administration Boston Healthcare SystemBostonMassachusetts; ^3^ Department of MedicineAlbert Einstein College of MedicineBronxNew York; ^4^ Department of Nutrition SciencesUniversity of Alabama School of MedicineBirminghamAlabama; ^5^ Department of GeneticsGeisel School of MedicineDartmouth UniversityHanoverNew Hampshire; ^6^ Departments of Genetics and BiologyUniversity of PennsylvaniaPhiladelphiaPennsylvania; ^7^ Dipartimento di Sanità Pubblica e Malattie InfettiveSapienza Università di RomaRomeItaly; ^8^ Centre National de Recherche et Formation sur le Paludisme, Ministère de la SantéOuagadougouBurkina Faso; ^9^ Hematology LaboratoryHôpital Saint‐LouisParisFrance; ^10^ Institut National de Recherche en Santé PubliqueBamakoMali; ^11^ Department of Molecular and Experimental MedicineThe Scripps Research InstituteLa JollaCalifornia

**Keywords:** Africa, amyloidosis, slave trade, transthyretin

## Abstract

**Background:**

Transthyretin (TTR) pV142I (rs76992529‐A) is one of the 113 variants in the human *TTR* gene associated with systemic amyloidosis. It results from a G to A transition at a CG dinucleotide in the codon for amino acid 122 of the mature protein (TTR V122I). The allele frequency is 0.0173 in African Americans.

**Methods:**

PCR‐based assays to genotype 2767 DNA samples obtained from participants in genetic studies from various African populations supplemented with sequencing data from 529 samples within the 1000 Genomes Project.

**Results:**

The rs76992529‐A variant allele was most prevalent (allele frequency 0.0253) in the contiguous West African countries of Sierra Leone, Guinea, Ivory Coast, Burkina Faso, Ghana, and Nigeria. In other African countries, the mean allele frequency was 0.011.

**Conclusions:**

Our data are consistent with a small number of founder carriers of the amyloidogenic *TTR* V122I (p.Val142Ile) allele in southern West Africa, with no apparent advantage or disadvantage of an allele carrying newborn reaching adulthood. In U.S. African Americans, the allele represents a significant risk for congestive heart failure late in life. If clinical penetrance is similar in African countries with high allele frequencies, then cardiac amyloidosis could also represent a significant cause of heart disease in the elderly in those populations.

## Introduction

Despite the predominant role of genomics, genome‐wide association studies, and deep sequencing technology in modern genetics, the study of well‐defined single base changes causing clinical disease in large populations remains important, particularly as therapies become available for such diseases. Furthermore, the disease‐associated variants can sometimes provide useful information regarding their evolutionary effect and the history of the carrier populations.

Transthyretin (TTR) is a homotetrameric, nonglycosylated serum and cerebrospinal fluid protein carrier of thyroxine and retinol‐binding protein charged with retinol (Buxbaum 2007). Each TTR monomer contains 127 amino acids. It is synthesized in the liver, choroid plexus, retinal epithelium, pancreas, and neurons (Buxbaum 2007; Li et al. 2011). It is a systemic carrier for thyroxine (T4) and retinol‐binding protein charged with retinol and has a critical function in transporting T4 into the central nervous system during a relatively narrow developmental window. Studies of homozygous *Ttr* knockout mice show significant structural disruption in the hippocampus with a quantitative deficit in neuroblast number in the supraventricular zone and neuronal loss in the CA3 region (Buxbaum et al. 2014). These are accompanied by deficits in spatial learning in adult mice (Sousa et al. 2004). It has been hypothesized that the phenotype is developmental. Neuronally synthesized TTR has been shown to behave as a stress protein, protective in transgenic murine models of human A*β* deposition with increased synthesis in human Alzheimer's disease (Wang et al. 2014).

The protein is encoded by a single‐copy gene (*TTR*) (NCBI reference sequence: NC_000018.10) on human chromosome 18 (Wallace et al. 1985). The gene is well conserved in humans with few variants relative to genes encoding other serum proteins and genes of the same size (Abecasis et al. 2010). A single protein polymorphism, a substitution of a serine for glycine at position 6 in the mature protein, occurs in approximately 12% of subjects of European descent (allele frequency 0.06) and to date seems unassociated with disease (Jacobson et al. 1995). Of 70 Africans screened, none were shown to carry this mutation, but it occurs in 2.5% of African Americans (Jacobson et al. 1995). The African American/White American allele frequency ratio of 0.0125/0.06 (0.21) is consistent with the mean estimate of admixture (0.17 ± 0.063) of European and African genes found in several studies across the U.S. population (Mersha and Abebe 2015). There is an additional rare coding sequence variant *TTR* Thr119Met (p.Thr139Met), which is kinetically very stable and has been reported to be associated with increased longevity in a large Danish population study (Hornstrup et al. 2013). The explanation for the longevity effect is unclear. Despite a significant number of large scale population surveys searching for variants, there has been no reported instance of a human completely lacking TTR.

To date there have been 123 amino acid substitutions, one deletion and one synonymous base substitution reported for the transthyretin protein and its encoding gene. Twelve have not been associated with tissue‐compromising TTR deposits, while in one instance the clinical amyloidogenicity has not been established. The amino acid variants have been shown to result in decreases in either the thermodynamic or kinetic stability of the tetramer and cause human disease (OMIM 176300.0009) (http://www.amyloidosismutations.com/mut-attr.php). The variants produce autosomal dominant diseases in which the tetramer dissociates into its constituent monomers that subsequently misfold and aggregate forming tissue oligomers and fibrils (Johnson et al. 2012). The aggregates are cytotoxic and may also compromise function on a displacement basis, depending on the target organ (Reixach et al. 2004). Most TTR variants have been described in only one or a few kindreds (Rowczenio et al. 2014). The most common variants associated with disease are pV50M (TTR V30M) and pV142I (TTRV122I). Both arose from transitions at CpG dinucleotides, a mutational “hot spot” (Cooper and Youssoufian 1988), as did the normal population variants TTR pG26S (TTR G6S) and pT139M (TTR T119M). Among the amyloidogenic TTR mutations, TTR V122I is unusual for its overwhelmingly predominant occurrence in individuals of documented African descent.

The heart is the major organ showing amyloid deposition in the pV142I (TTR V122I) carriers. It was first described in 1988 in an African American man with cardiac amyloidosis who was homozygous for the G→A transition at rs76992529 (Gorevic et al. 1989; Jacobson et al. 1990). Many other reports have confirmed the association of the variant with cardiac amyloidosis in elderly African Americans and it is now recognized as a cause of heart failure when the allele is present in either the heterozygous or homozygous state (Jacobson et al. 1990; Nichols et al. 1991). Population studies have determined that the prevalence of the *TTR* rs76992529‐A allele in African Americans is 0.0173 (Jacobson et al. 2015). A small number of kindreds carrying the allele without identifiable African ancestry have been reported (Gillmore et al. 1999; Asl et al. 2001; Ammirati et al. 2012) (Cappelli et al. 2015; Damy et al. 2015). The amyloidogenic allele is an infrequent but valid ancestry informative marker. It appears that in the heterozygous state the allele is clinically silent until the seventh decade of life or later. Homozygous individuals develop disease about 10 years earlier (Reddi et al. 2014). Given the late age of clinical impact, the allele is likely to be evolutionarily neutral.

One previous study reported the allele frequency in small selected African populations: it was found in 1/55 South African Zulus, 0/34 South African Xhosas, and 0/9 Nigerians (Afolabi et al. 2000). Since the African American population is largely descended from African slaves brought to the western hemisphere from West Africa in the 16th to 19th centuries (Curtin 1969), we elected to investigate its origin(s) by determining the frequency of the *TTR* V122I allele in various modern African cohorts.

## Methods

### Ethical compliance

De‐identified DNA samples from 14 modern African countries were obtained with informed consent by investigators studying the genetics of a variety of diseases not known to be related to TTR and laboratories interested in the nature of genetic differences among African populations (Table 1). The samples from the Gambia obtained specifically for this study were approved by the Gambia ethics committee of the Medical Research Council (MRC). The samples were provided to us with information about the country and, in some cases, the village and/or tribe of origin. We have also included data from 529 African DNA samples analyzed and reported in the “1000 Genomes Project” (Abecasis et al. 2010).

**Table 1:** Frequency of TTR V122I in African populations

Country/Region	Population source	V122I/Total alleles	Allele prevalence (sample)	V122I alleles	Total alleles	Allele prevalence (country)
Burkina Faso	Modiano et al. (1999)			**3**	**120**	**0.025**
Ghana	**Total**			**61**	**2424**	**0.025**
	Williams et al. (2000)	13/528	0.025			
	Present study	48/1896	0.026			
Guinea	Zimmerman et al. (1995)			**2**	**56**	**0.036**
Ivory Coast	Zimmerman et al. (1995)			**3**	**82**	**0.037**
Sierra Leone	**Total**			**31**	**1174**	**0.026**
	Zimmerman et al. (1995)	6/100	0.060			
	Jackson et al. (2005)	22/904	0.024			
Mende	Abecasis et al. (2010)	3/170	0.018			
Nigeria	**Total**			**12**	**570**	**0.021**
	Vulliamy et al. (1991)	0/122	<0.008			
	Afolabi et al. (2000)	0/18	<0.055			
Esan	Abecasis et al. (2010)	6/198	0.030			
Yoruba	Abecasis et al. (2010)	6/232	0.026			
Total, high‐prevalence area				**112**	**4426**	**0.0253**
Gambia	**Total**			**8**	**636**	**0.013**
	Cooke et al. (2004); Sirugo et al. (2004)	5/410	0.012			
	Abecasis et al. (2010)	3/226	0.013			
Guinea‐Bissau	Zimmerman et al. (1995)			**1**	**78**	**0.013**
Mali	Kalidi et al. (1988)			**1**	**130**	**0.008**
Senegal	Zimmerman et al. (1995)			**1**	**132**	**0.008**
Tanzania	Henn et al. (2008)			**3**	**254**	**0.012**
Cameroon	Henn et al. (2008)			**3**	**230**	**0.013**
Kenya				**1**	**264**	**0.004**
Luhya (Kenya)	Abecasis et al. (2010)	1/232	0.009			
Other Kenya		0/32	<0.030			
South Africa				**3**	**360**	**0.008**
Xhosa	Afolabi et al. (2000)	0/68	<0.015			
Zulu	Afolabi et al. (2000)	1/110	0.009			
Xhosa	Rousseau et al. (1985)	2/182	0.011			
Total, outside high‐prevalence area				**21**	**2084**	**0.010**

The *TTR* rs76992529‐A allele was identified using PCR‐based assays developed in our laboratory (Jacobson 1992) (Alexander et al. 2004) except in the data available from the 1000 Genomes Project in which complete DNA sequencing was performed (Abecasis et al. 2010).

## Results

**Figure 1:**
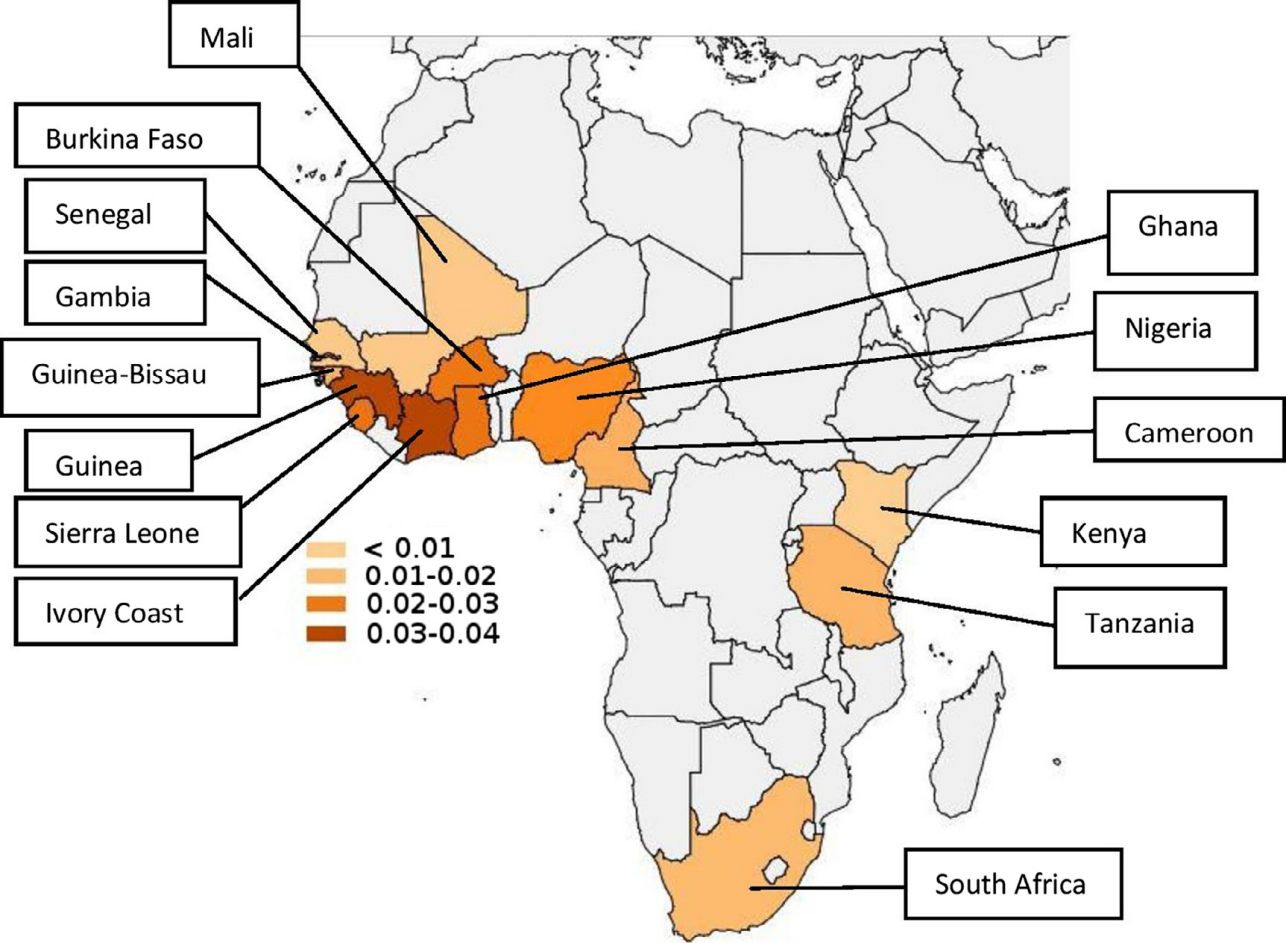
Distribution of the TTR V122I allele in Africa (see Table 1). The allele frequencies are indicated by color. Populations from the countries colored light gray were not analyzed.

Of the roughly 2900 samples tested in our laboratories, successful results were obtained in 2767. The prevalence of the *TTR* allele encoding pV142I was highest in six contiguous countries of West Africa, ranging from Nigeria in the east to Guinea in the west (Table 1 and Fig. 1). The overall allele frequency in this area was 0.0253, with each of the individual countries having an allele frequency of at least 0.021. In contrast, the allele frequencies in the other 10 African countries studied ranged from 0 to 0.013, with an overall frequency in those countries of 0.011. The 0.0253 frequency in the high‐prevalence area was statistically different from the frequency in samples studied from outside this area (Table 1, Fig. 1) (*P* = 0.0001) (two‐tailed *Z* test).

## Discussion

It is striking that within the African countries examined in this study, the frequency of the *TTR p*V142I (TTR V122I)‐encoding allele was significantly higher in one contiguous geographic area of West Africa (overall allele prevalence 0.0253, ranging from 0.021 to 0.037 in the individual countries studied) than elsewhere in Africa (allele prevalence 0.010–0.011). The overall allele prevalence of 0.0253 in the high‐prevalence area indicates that about 5.1% of the population is expected to be heterozygous for the variant allele. While the allele prevalence is significantly lower in other African regions, it was present wherever we were able to sample throughout sub‐Saharan and western Africa (Fig. 1, Table 1).

Since the Ghanaian samples were obtained from 948 newborns and 264 adults, we could compare the allele frequencies in two different age groups from the same population (as determined in a single geographic area). The allele frequency was nearly identical in the newborns (49/1896; 0.026) and the adults (13/528; 0.025) from Accra. These data, coupled with the late age of onset of the allele‐associated disease, support the conclusion that the allele does not influence survival to adulthood and is unlikely to be under positive or negative selective evolutionary pressure. The allele prevalence in African Americans is close to that in the modern West African countries that were the geographic center of the slave trade suggesting that the gene was present in similar proportions in the population ancestral to modern African Americans and had little effect on survival in North America (Curtin 1969).

A recent analysis of the African samples from the “1000 Genomes Project” showed that the G to A transition responsible for the variant amino acid occurred on five different haplotypes (Polimanti et al. 2014). Four of these differed from the haplotype containing the ancestral allele at 12 different positions within the confines of the *TTR* gene and were identical at all but one base, indicating a single founder haplotype that acquired noncoding mutations after the appearance of the amyloidogenic *TTR* V122I allele. The second potential founder haplotype differed from the ancestral haplotype at 13 positions and from the other possible founder haplotype at 12 positions, five of which it shared with the ancestral allele suggesting a second founding event or a more complex set of alterations in the gene after the initial appearance of rs76992529. In any case, despite the origin of the allele at a CpG dinucleotide, a so‐called genetic “hot spot,” the number of founder haplotypes is small. Previous haplotype analyses in African American carriers of the allele also suggested a small number of founders in the descendants of the original slave population (Jacobson et al. 1998).

Genetic, epigenetic, and environmental factors have been proposed as being responsible for variation in the clinical penetrance, that is, clinically important TTR amyloid deposition, of any *TTR* mutation. The first‐described and most extensively studied disease‐associated *TTR* variant, TTR pV50M (TTR V30M) was identified in Portugal, then Sweden, and subsequently Japan, and has since been found globally (Andrade 1952; Araki et al. 1968; Andersson 1976). The age of the allele has been estimated to be 26 generations (750 years) in Portugal, 15 generations (375 years) in Sweden (Zaros et al. 2008), and perhaps older in Italy (Iorio et al. 2014). Analysis of intronic haplotypes revealed multiple founders in Japan, but single founders in Portugal and Sweden (Yoshioka et al. 1989; Zaros et al. 2008). More recent studies have identified multiple haplotypes across Europe as well (Reilly et al. 1995; Soares et al. 2004). There are significant differences in clinical penetrance of TTRV30M among the carriers in different populations, particularly with respect to age of onset (Koike et al. 2002) (Hellman et al. 2008). We have suggested that disease penetrance is conditioned by epistatic interactions with other genes, the products of which can influence the stability of the tetramer (Soares et al. 2005). Support for that idea has recently been reported independently (Santos et al. 2015). Others have hypothesized that variation at transcription factor binding sites within populations resulting in different transcription levels presumably increased, resulting in increased serum TTR concentrations, could also be responsible for different degrees of clinical penetrance (Polimanti et al. 2014). While there are no available data regarding hepatic TTR transcription in different carriers of the same amyloidogenic allele, we think this unlikely since serum TTR concentrations tend to be lower rather than higher in carrier subjects even before there is detectable tissue deposition.

Studies of polymorphisms in a microRNA binding site in the promoter region of *TTR* V30M (pV50M) carriers in Sweden, while initially suggesting an explanation for the apparent late onset in Swedish carriers, could not be confirmed (Olsson et al. 2010; Norgren et al. 2012). In individuals carrying *TTR* V30M, it appears that the parent of inheritance (POI) has a significant effect on disease penetrance, carrier sons of carrier mothers having earlier onset, and more severe disease (Sousa et al. 1991). Some studies have suggested that the POI is related to mitochondrial genotype; however, it does not appear that the entire effect can be attributed to mitochondrial inheritance (Olsson et al. 2008; Bonaiti et al. 2010). One can assume that the same phenomena will prevail for disease related to all the amyloidogenic TTR mutations including pV142I (TTRV122I).

The mechanism of TTR amyloid formation is similar regardless of the nature of the specific mutation, that is, dissociation of the TTR tetramer due to reduced thermodynamic or kinetic stability, which allows the released monomers to misfold and aggregate (Johnson et al. 2012). It is likely that genetic or epigenetic factors that influence the development of disease operate in a similar manner regardless of the specific mutation, having a greater or lesser effect depending on the stability of the variant.

Among the samples from Sierra Leone, Cameroon, Tanzania, Burkina Faso, Nigeria, and South Africa, the variant allele was found in samples from multiple tribal origins and language groups (Tables 1 and 2). Our sample sizes were not large enough to determine whether the differences in allele frequencies among tribes were statistically significant. In the absence of larger sample sizes and haplotype analysis in those individuals we cannot determine whether these represented rare individual founders or migrants into the groups from areas in which the allele was present at higher frequencies.

**Table 2:** TTR V122I allele prevalence in specific tribes in Tanzania, Cameroon, and Sierra Leone

Tribe	TTR V122I	TTR V122V	Allele frequency
Tanzania and Cameroon			
Turu	0	56	<0.018
Sandawe	1	61	0.016
Burunge	2	50	0.038
Maasai	0	58	<0.017
Bakola	0	56	<0.018
Bamoun	1	55	0.018
Zime	2	46	0.042
Sierra Leone			
Creole	4	70	0.054
Shabro	1	1	1
Mende	9	236	0.038
Temne	3	195	0.015

The similar allele frequencies in newborn and adult Ghanaians suggest that the *TTR* V122I (pV142I) allele does not confer an advantage or disadvantage in reaching adulthood in the Ghanaian environment. Similarly, the 0.0173 allele frequency in U.S. African Americans, allowing for an average admixture of 17% with European genes, is consistent with the 0.0253 frequency in Africans in the high‐prevalence area, and suggests that there is little selection against allele carriers in the United States environment. Hence, current allele frequencies across Africa may reflect its distribution at the time of the Atlantic slave trade from the 15th to 19th centuries A.D.

The *TTR* V122I (pV142I) allele frequencies are highest in the non‐Bantu‐speaking Niger‐Kordofanian‐derived populations from Sierra Leone, Ghana, Cote d'Ivoire, etc. Broad genetic studies have indicated that this is the ancestry that is mostly highly represented in African Americans (Tishkoff et al. 2009; Bryc et al. 2010). Interestingly, the frequencies of the TTR V122I (pV142I) allele are low in the countries immediately adjacent to the north and west (Gambia, Guinea‐Bissau, Mali, and Senegal) as well as those to the south and east.

The major population movement as judged initially by language patterns across Africa was the so‐called Bantu expansion from 1000 B.C. to 500 A.D. During that period Bantu language‐speaking people spread, apparently in several stages, from a relatively small area in what is now the southeastern border of Nigeria and Cameroon, south and eastward, dispersing throughout the southern half of the continent. In our study, the lower allele frequency in the Cameroonian sample than in the Nigerian cohorts and the considerable variation in the frequency among the various samples from Nigeria could be consistent with a relatively indistinct western border of the Bantu region in the context of modern national boundaries. The observation that the current frequency of the *TTR* V122I (pV142I) allele is three times greater in coastal West Africa than in the other parts of the continent that we were able to sample is open to several interpretations. It is possible that the allele may be older than the expansion, and originated in a non‐Bantu‐speaking, western coastal population that was unaffected by the migration, and that it has persisted in the local populations because it is evolutionarily neutral or under weak purifying selection. Alternatively, the allele may have appeared after the Bantu expansion with the lower allele frequency among the Bantu speakers representing small groups of later migrating founders.

Based on studies of over 12,000 DNA samples, the prevalence of the allele encoding TTR pV142I allele in African Americans is known to be 0.0173 (Jacobson et al. 2015). Furthermore, it is estimated that modern African Americans have an average of 17% genetic admixture with groups with non‐African ancestry, although there is substantial variation among individuals and in different geographic regions in the United States (Kostrikis et al. 1998; Halder et al. 2009, 2012; Tishkoff et al. 2009; Bryc et al. 2010, 2014; Murray et al. 2010; Tandon et al. 2011; Bhatia et al. 2014; Mersha and Abebe 2015). Thus, based on an inferred population without significant admixture one can estimate that the average variant allele prevalence in the African ancestors of modern African Americans would be 0.0173/0.83 or 0.021, not significantly different from the combined observed frequency in the high‐prevalence area (Ghana, Sierra Leone, Guinea, Burkina Faso, Cote d'Ivoire, and Nigeria), that is, 0.025.

Is this calculation consistent with what is known about the Atlantic slave trade? The available historical data indicate that 50–70% of African slaves brought to North America came from areas corresponding to the modern African countries included in our designation “West Africa” plus the immediately adjacent countries of Togo and Benin, which were not represented among our samples (whereas higher percentages of slaves from other areas, such as modern‐day Congo and Angola, for which we do not have allele frequency data, may have been taken, in higher numbers, to South America) (Curtin 1969). Correlating historical records of the slave trade with modern African political boundaries is imprecise, because of gaps in the available data and the breakdown of Africa into geographic areas in the 16th to 19th centuries (e.g., Bight of Benin [including the southern coasts of Ghana, Togo, Benin, and the western coast of Nigeria], Bight of Biafra [including the presently defined southern coast of Nigeria, Cameroon, Equatorial Guinea, and the northern coast of Gabon], Windward Coasts, Gold Coast [Ghana], etc.) do not precisely correspond to modern African countries. Thus, our data and model seem consistent with the historical record of the Atlantic slave trade.

We conclude that the prevalence of the *TTR* V122I allele in Africa ranges from 0.037 around the area presumed to include most ancestors of African Americans to 0.010 in African countries less likely to be highly represented among the slave population brought to North America. Haplotype data from the 1000 genomes project are consistent with perhaps two ancient founder haplotypes carrying the amyloidogenic rs76992529‐A I allele (Polimanti et al. 2013). Future population‐based surveys of this allele focused on smaller regions and tribal studies within the region we have defined as “southern West Africa” plus the adjacent areas of Togo and Benin might reveal small areas with an even higher prevalence. Such studies would be of interest for both genetic purposes, and for the purposes of ultimately providing optimal care for patients from these areas. As life expectancy in the region increases, TTR V122I (pV142I) carriers may represent a cohort of individuals at substantial risk of developing clinically significant cardiac amyloidosis with aging.

## Conflict of Interest

None of the authors has financial or other interests which would compromise their objectivity in either performing or interpreting the analyses reported.
